# Bone defect development in experimental canine peri-implantitis models: a systematic review

**DOI:** 10.1186/s13643-022-02075-3

**Published:** 2022-09-21

**Authors:** A. Solderer, M. de Boer, D. B. Wiedemeier, M. Solderer, C. C. Liu, P. R. Schmidlin

**Affiliations:** 1 Clinic of Conservative and Preventive Dentistry, Division of Periodontology and Peri-implant Diseases, Center of Dental Medicine, University of Zurich, 8032 Zurich, Switzerland; 2 Private Practice, 39100 Bolzano, Italy; 3 Statistical Services, Center of Dental Medicine, University of Zurich, Zurich, Switzerland

**Keywords:** Bone defects, Dental implant, Dogs, Ligature, Peri-implantitis, Review

## Abstract

**Purpose:**

To provide a systematic overview of preclinical research regarding bone defect formation around different implant surfaces after ligature-induced peri-implantitis models in dogs. Two focused questions were formulated: ‘How much bone loss can be expected after a certain time of ligature induced peri-implantitis?’ and ‘Do different implant types, dog breeds and study protocols differ in their extent of bone loss?’

**Materials and methods:**

A systematic literature search was conducted on four databases (MEDLINE, Web of Science, EMBASE and Scopus). Observations, which consisted of bone defects measured directly after ligature removal in canine models, were included and analysed. Two approaches were used to analyse the relatively heterogeneous studies that fulfilled the inclusion criteria. First, separate simple linear regressions were calculated for each study and implant surface, for which observations were available across multiple time points. Second, a linear mixed model was specified for the observations at 12 weeks after ligature initiation, and assessing the potential influencing factors on defect depth was explored using lasso regularisation.

**Results:**

Thirty-six studies with a total of 1082 implants were included after. Bone loss was determined at different time points, either with clinical measurements radiographically or histologically. Different implant groups [e.g. turned, sand-blasted-acid-etched (SLA), titanium-plasma-sprayed (TPS) and other rough surfaces] were assessed and described in the studies. A mean incremental defect depth increase of 0.08 mm (SD: −0.01–0.28 mm) per week was observed. After 12 weeks, the defect depths ranged between 0.7 and 5 mm. Based on the current data set, implant surface could not be statistically identified as an essential factor in defect depth after 12 weeks of ligature-induced peri-implantitis.

**Conclusion:**

Expectable defect depth after a specific time of ligature-induced peri-implantitis can vary robustly. It is currently impossible to delineate apparent differences in bone loss around different implant surfaces.

**Supplementary Information:**

The online version contains supplementary material available at 10.1186/s13643-022-02075-3.

## Background

Implantology is a growing field in dentistry, and an increasing number of dental implants are being placed every year [1]. Promising outcomes have been shown in modern implant therapy. Nevertheless, these procedures are not immune to biological complications. Similarly to periodontal inflammation, peri-implant diseases are etiologically induced by pathogenic biofilm accumulation. Inflammatory lesions affecting only the mucosa are defined as peri-implant mucositis, while advanced inflammations, resulting in bone loss, are defined as peri-implantitis. The clinical picture is characterised by bleeding, suppuration on probing or both combined with an increased probing depth and progressing bone loss [2].

Over 16% of patients with dental implants suffer from peri-implantitis [3], and it occurs in 22% (CI: 14–30%) of all implants placed [4], representing the primary cause of implant failure [5]. A critical aspect of everyday patient implant management is highlighted in such a scenario. Similar bacterial compositions are associated with peri-implantitis and periodontitis [6].

Dental implants affected by peri-implantitis can be treated with different approaches. However, no specific treatment protocol seems to show conceptually distinct advantages, leading to a need for further peri-implantitis biology research and treatment-related strategy solutions [7].

Research in animal models represents a standard preclinical setting. The association between bacterial biofilm and the pathogenesis of periodontitis was first described in teeth in rats by Rovin et al. in 1966 [8]. In 1973, the model was first applied to beagle dogs in periodontal research [9]. Inflammatory lesions were induced by terminating the plaque control regimen and using submucosal ligatures made of different materials around the implant neck, allowing biofilm formation [10]. In 1992, Berglundh et al. first transferred the model to peri-implantitis research [11]. Since then, many studies following these basic principles have been published.

The canine model rapidly became one of the most well-established animal models to study peri-implantitis due to the similarity of periodontal anatomy and inflammation development between dogs and humans [12]. Schwarz et al. [13] described the saucer-shaped circumferential peri-implant defect (Class Ie) as the most commonly encountered defect morphology, with 55% in humans and 85% in animals [13]. With the application of ligatures, the above-described models have been introduced to promote tissue breakdown in shorter periods than in earlier models, which had to prepare experimental animals over the years [9]. Despite a strong consensus that marginal bone resorption is caused by biofilm accumulation, a possible influence of other factors, such as the trauma of ligature insertion or related immunological reactions, cannot be excluded [14].

Despite many studies following the principles described above, no standardised concept with predefined parameters regarding time, materials, implant surfaces and expectable defect depths in this model was established. This scenario resulted in a heterogeneous field of hardly comparable preclinical research.

Therefore, in this systematic review, we aimed to provide an overview of preclinical peri-implantitis studies in dogs and to assess the expectable formation of bone defects after ligature-induced peri-implantitis. Furthermore, the influence of different implant surfaces on the extent of bone loss was also investigated.

## Materials and methods

This systematic review followed the PRISMA statement for transparent reporting of systematic reviews and meta-analyses [15].

### Focused questions

Two focused questions were formulated: ‘How much bone loss can be expected after a certain time of ligature induced peri-implantitis?’ and ‘Do different implant types, dog breeds and study protocols differ in their extent of bone loss?’ These focused questions concern the canine as a model and measurements taken immediately after ligature removal.

- Primary outcome:

Bone loss (defect depth measured in mm) is expected after different periods of ligature-induced peri-implantitis.

- Secondary outcomes:

Bone loss depends on different implant surfaces, diameters, dog breeds, ligature material, ligature changing protocol and measurement of defect depths.

### Search strategy

A systematic literature search was conducted on different databases: MEDLINE, Web of Science, EMBASE and Scopus. Only English studies were included. Articles published up to and including December 2020 were searched. The detailed search protocol for the MEDLINE database can be found in Additional file 1.

### Eligibility criteria

The following inclusion criteria were defined for this systematic review: (1) implant studies conducted in a canine model, (2) ligature-induced peri-implantitis and (3) clinical, radiographical or histological evaluation of bone loss in millimetres (mm) around infected implant tissue after a specific period. The exclusion criteria were as follows: (1) human studies or animal studies not performed on dogs, (2) studies with interventions influencing the formation of bone defects, (3) studies without ligature placement and (4) studies not providing raw data on defect depths after contacting authors.

### Screening and selection

The titles and abstracts of all reports identified through the electronic searches were screened by two different authors (A.S. and M.d.B.). A full description was obtained for studies that met the inclusion criteria and those lacking sufficient data to decide by reading their titles and abstracts. Cohen’s kappa for the title and abstract screening was 0.8. Disagreements were discussed between the two authors and the senior author (P.S.) until a mutual agreement was reached.

### Data extraction

The data were extracted by the same two authors (A.S. and M.d.B.): author, release date, defect depth (bone loss in mm), standard deviation, the period between ligature placement and defect depth measurement (the active period in weeks), the period after ligature removal until further measurement of bone loss (passive period in weeks), evaluation method (clinical/radiographical/histological), implant type (e.g. turned, sand-blasted-acid-etched (SLA), titanium-plasma-sprayed (TPS) and other rough surfaces), number of implants inserted in the study, number of dogs, dog breed, dimensions of the implant (in mm) and ligature change. If no raw data were available, the authors of the studies were contacted. If additional data were not received, the studies were excluded.

### Data preparation

Data were collected and assessed according to three different aspects. First, studies were evaluated overall regarding key points for graphical and descriptive analysis of defect formation over time: authors, study date, defect depth and time of active tissue breakdown. The variable period of ongoing passive tissue breakdown after ligature removal was excluded from the statistical analysis. Furthermore, 40 observations (from seven different studies) not providing the raw data needed were removed from the data set. Second, data for the separate linear regression models assessing the influence of different implant surfaces (four groups: turned, SLA, TPS and other rough surfaces) over time of tissue breakdown in bone defect depths were collected. Finally, data of critical points that may influence defect depth at 12 weeks: implant surface (four groups: turned, SLA, TPS and other rough surfaces), implant diameter, ligature type, ligature change, measurement method and dog breed were assessed. All implants were assigned into two different diameter groups: (1) implants with a diameter lower than 3.5 mm were referred to as ‘narrow’, while (2) implants from and above 3.5 mm represented the ‘regular’ group. The measurement method (how defect depth was measured) was categorised into clinical, histological and radiographic approaches.

### Data analyses

The entire dataset was analysed exploratorily using different approaches from the statistical toolbox. All available data were visualised using time-series plots and boxplots. Since meta-analytical modelling was not suitable, two alternative modelling approaches were followed because not enough observations followed comparable experimental conditions.

First, all studies investigating implant type at multiple time points were identified to examine the effect of implant application over time on defect depth. Separate simple regression models were calculated for each study, and implant type combination with an active period of peri-implantitis induction was the only explanatory factor.

Then, a linear mixed model was fitted to the observations at 12 weeks, in which the possible influence of the collected factors (e.g. implant surface, implant diameter, ligature type, ligature change, measurement method and dog breed) was checked. The time point of 12 weeks was chosen because it was the time for which most data could be collected and, thus, was most promising for disentangling different impacts on defect depth. The dependence of the observed defect depth on the experimental conditions in each study was accounted for by a random intercept in the model. Variable selection on the fully specified model, including all possible explanatory variables (all collected factors), was then conducted using the lasso method with cross-validation. The Bayesian information criterion and the Akaike information criterion were also used to deduce the regularisation parameter.

All statistical analyses and plots were computed using the statistical software R [16]. The packages used in the analyses were tidyverse [17] and glmmLasso [18].

## Results

### Literature search

**Fig. 1:**
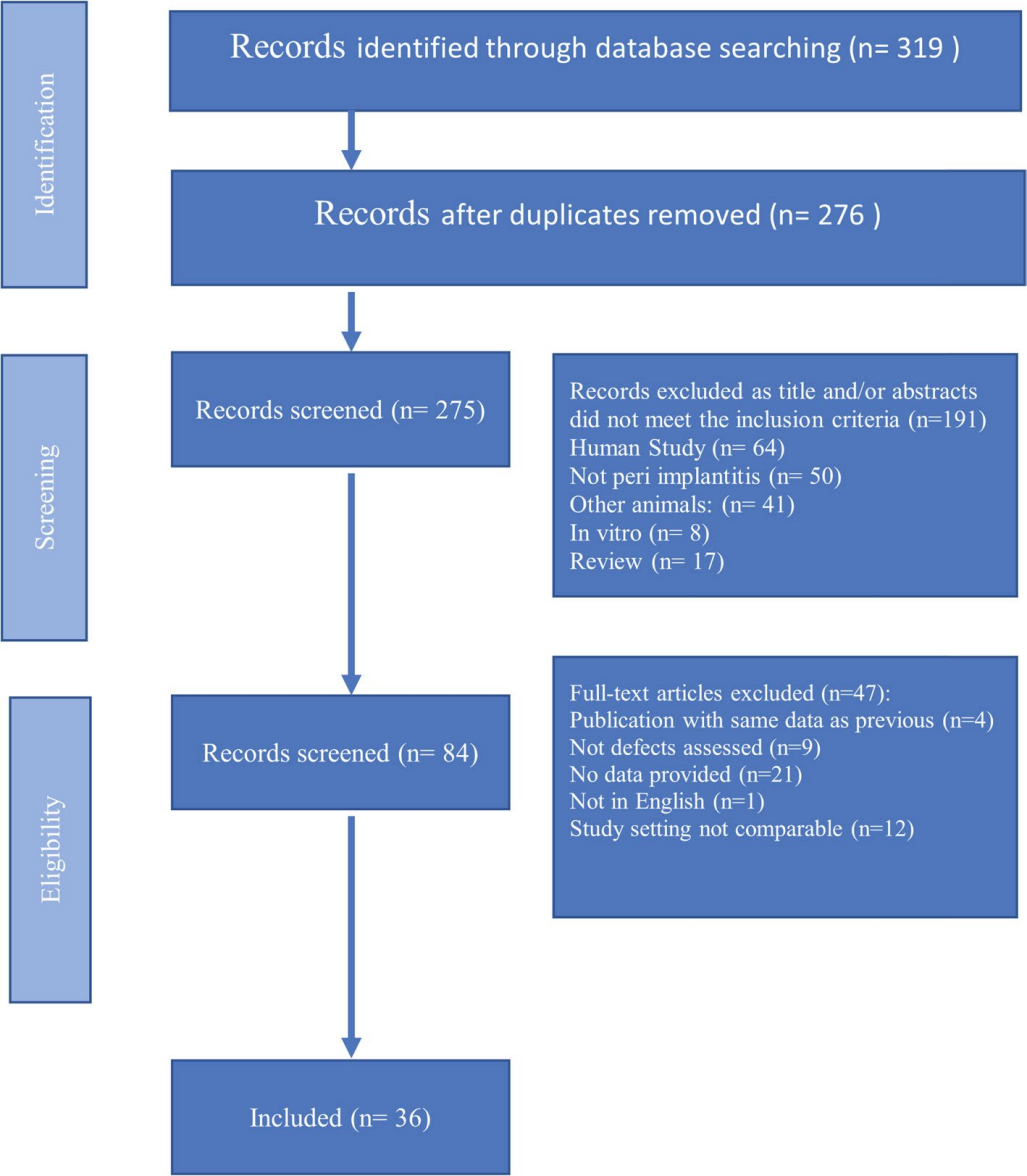
PRISMA flowchart

The study selection process is summarised in Fig. 1. A total of 319 papers were identified through database searches. One of these papers was added through a hand search. Around 275 articles remained after duplicates were removed from more than one database. The titles and abstracts of those 275 papers were screened by two reviewers (A.S. & M.d.B), and 191 were excluded for being related to another topic or not meeting the inclusion criteria. Of the 84 studies that had their full text screened, 47 articles were excluded for not assessing the defect depth, not providing relevant data, not being written in English or having a non-comparable study setting (e.g. applying drugs). Finally, 36 studies were included in this systematic review.

### Studies characteristics

An overview of the included studies and the data assessed are given in Table 1. A total of 1082 implants were included in this systematic review. For reasons of transparency, the implants were divided into the following groups: turned (*n* = 216), SLA (*n* = 250), TPS (*n* = 202) and the remaining were gathered into the group ‘other rough surfaces’ (*n* = 414).

**Table 1:** Overview of the included studies

Author (year)	Number of dogs	Number of implants	Active peri-implantitis induction (weeks)	Implant surface type	Method of measurement	Ligature material and change of ligature	Implant diameter (mm)	Dog breed
Albouy et al., 2008 [19]	6	5	12	Turned	Clinical	Silk, ligature change	3.25 × 10	Labrador
		6		Other rough surfaces			3.5 × 11	
		6		SLA			3.3 × 10	
		5		Other rough surfaces			3.3 × 10	
Albouy et al., 2012 [20]	5	8	10	Turned	Radiographic	Cotton, ligature change	3.3 × 10	Labrador
		8		Other rough surfaces			3.3 × 10	
Almohandes et al., 2019 [21]	6	12	12	TPS	Radiographic	Cotton, ligature change	3.6 × 11	Labrador
		12		Turned			3.6 × 11	
Battula et al., 2015 [22]	4	4	18, 24, 30, 38	Turned	Clinical	Silk, no ligature change	4.1 × 13	Hound dog
		4		Other rough surfaces			4.1 × 13	
		4		Turned			4.1 × 13	
		4		Other rough surfaces			4.1 × 13	
Berglundh et al., 2007 [23]	5	15	16	SLA	Radiographic	Cotton, ligature change	3.3 × 8	Beagle
		15		Turned			3.3 × 8	
Carcuac et al., 2013 [24]	5	10	10	Turned	Radiographic	Cotton, ligature change	3.3 × 10	Labrador
		10		Other rough surfaces			3.3 × 10	
Carcuac et al., 2015 [25]	6	6	9	Other rough surfaces	Radiographic	Cotton, ligature change	3.5 × 11	Labrador
		6		Other rough surfaces			3.5 × 11	
		6		Other rough surfaces			3.5 × 11	
		6		Other rough surfaces			3.3 × 11.5	
Deppe et al., 2001 [26]	6	60	12	TPS	Radiographic	Cotton, no ligature change	11m	Beagle
Godoy-Gallardo et al., 2016 [27]	5	10	8	Other rough surfaces	Radiographic	Silk, no ligature change	NA	Beagle
		10		Other rough surfaces			NA	
		10		Other rough surfaces			NA	
Grunder et al., 1993 [28]	10	10	4, 8, 12, 16, 20	Turned	Clinical	Cotton, ligature change	3.25 × 7	Beagle
		10		Turned			3.25 × 7	
		10		Turned			3.25 × 7	
		10		Turned			3.25 × 7	
Huang et al., 2015 [29]	6	6	12	Other rough surfaces	Clinical, radiographic	Cotton, no ligature change	3.5 × 8	Beagle
		6		Other rough surfaces			3.5 × 8	
		6		Other rough surfaces			3.5 × 8	
		6		Other rough surfaces			3.5 × 8	
Huang et al., 2018 [30]	6	6	12	TPS	Histological	Cotton, no ligature change	3.5 × 8	Beagle
		6		TPS			3.5 × 8	
		6		TPS			3.5 × 8	
		6		TPS			3.5 × 8	
Lang et al., 1994 [31]	5	12	16	TPS	Clinical, histological	Silk, additional ligatures	2.8 × 6	Beagle
		12		TPS				
Madi et al., 2014 [32]	4	12	16	Turned	Clinical	Silk, ligature change	3.3 × 10	Beagle
		12		SLA			3.3 × 10	
		12		HA			3.3 × 10	
		12		TPS			3.25 × 10	
Madi et al., 2016 [33]	4	12	16	Turned	Clinical	Cotton, ligature change	3.3 × 10	Beagle
		12		SLA			3.3 × 10	
		12		HA			3.3 × 10	
		12		TPS			3.25 × 10	
Martins et al., 2005 [34]	6	9	8	Turned	Clinical	Cotton, additional ligatures	3.75 × 10	Mongrel dog
		9		TPS			4.1 × 10	
		9		HA			3.75 × 10	
		9		Other rough surfaces			3.75 × 10	
Marinello et al., 1995 [35]	5	4	4	TPS	Histological	Cotton, ligature change	3.75 × 10	Labrador
Martinez et al., 2014 [36]	5	10	12	Other rough surfaces	Histological	Cotton, ligature change	3.75 × 11.5	
		10		Other rough surfaces			3.75 × 11.5	
		9		Other rough surfaces			3.75 × 11.5	
Monje et al., 2018 [37]	6	18	3, 9	Other rough surfaces	Clinical	Silk, additional ligature	NA	Beagle
		18		Other rough surfaces			NA	
Monje et al., 2021 [38]	6	18	3, 6, 9	Turned	Clinical, radiographic	Silk, ligature change	NA	Beagle
		18		Other rough surfaces			NA	
Namgoong et al., 2015 [39]	5	10	5, 8, 12, 18, 23	Turned	Radiographic	Steel, no ligature change	3 × 8.5	Beagle
		10		SLA			3 × 8.5	
		10		TPS			3 × 8.5	
Park et al., 2017 [40]	4	8	12	SLA	Clinical	Cotton, change	3.5 × 8.5	Beagle
		8		SLA			3.5 × 8.5	
		8		SLA				
		8		SLA				
Park et al., 2020 [41]	4	8	12	SLA	Histological	Cotton, additional ligature	3.5 × 8.5	Beagle
Persson et al., 1999 [42]	4	8	16	TPS	Radiographic	Cotton, ligature change	3.3 × 8	Beagle
		8		TPS			3.3 × 8	
Sanz- Esporrin et al., 2019 [43]	12	18	12	SLA	Histological	Silk, ligature change	3.3 × 8	Beagle
		17		SLA			3.3 × 8	
Shi et al., 2015 [44]	6	6	12	Other rough surfaces	Clinical, radiographic	Dental floss, no ligature change	3.3 × 8	Beagle
		6		Other rough surfaces			3.3 × 8	
		6		SLA			3.3 × 9	
Solderer et al., 2020 [5]	12	7	8	SLA	Clinical	Cotton, no ligature change	3.3 × 8	Beagle
		9		SLA			3.3 × 8	
		9		SLA			3.3 × 8	
		8		SLA			3.3 × 8	
Solderer et al., 2021 [45]	4	24	8	SLA	Radiographic	Cotton, no ligature change	3.3 × 8	Beagle
Tillmanns et al., 1997 [46]	6	14	4, 8, 12	Turned	Clinical	Cotton, NA	4 × 10	Beagle
		14		Other rough surfaces			4 × 10	
		14		Cther rough surfaces			4 × 10	
Vigano et al., 2019 [47]	6	12	12	TPS	Radiographic	NA, ligature change	3.3 × NA	Beagle
		12		TPS				
Wetzel et al., 1999 [48]	7	11	16	SLA	Clinical	Silk, additional ligature	2.8 × 6	Beagle
		6		SLA			2.8 × 6	
		7		Turned			2.8 × 6	
Xu et al., 2016 [49]	6	12	12	Turned	Clinical, radiographic	Cotton, no ligature change	3.8 × 10	Beagle
		12		Turned				
Yang et al., 2018 [50]	6	8	2, 4, 6, 8, 10, 12	SLA	Clinical	NA, additional ligature	4.1 × 8	Labrador
Yoon et al., 2020 [51]	6	6	6	Other rough surfaces	Radiographic	Silk, no ligature change	3.6 × 8	Beagle
		6		Other rough surfaces			3.6 × 8	
		6		Other rough surfaces			3.6 × 8	
		6		Other rough surfaces			3.6 × 8	
Zitzmann et al., 2004 [52]	5	21	8	TPS	Radiographic	Cotton, ligature change	3.75 × 10	Labrador

The turned implant surfaces were included in around 14 studies and were also referred to as machined or polished. The SLA surface represented a sand-blasted, large grit, acid-etched implant surface and was classified as a rough surface. Fourteen studies were included in which SLA surfaces were examined. Implants with TPS surfaces were included in 12 studies. The group ‘other rough surfaces’ consisted of all other implant types and surfaces included in the studies, which did not fit the groups above. Finally, 18 studies in which the implants of this group were assessed were included in this group.

#### Defect formation over time

The presence of ligatures and increasing biofilm resulted in inflammation of the tissue surrounding the implant and bone loss, referred to as peri-implantitis. The so-called active breakdown period was when the ligature was placed around the implant neck [19]. After this active breakdown period, the ligatures were removed. Some studies continued measuring the bone loss that occurred in the following passive breakdown period after ligature removal [19, 23–25, 27, 35, 37, 39, 42, 52]. Only data concerning the ‘active breakdown’ progression were included in the analysis.

**Fig. 2:**
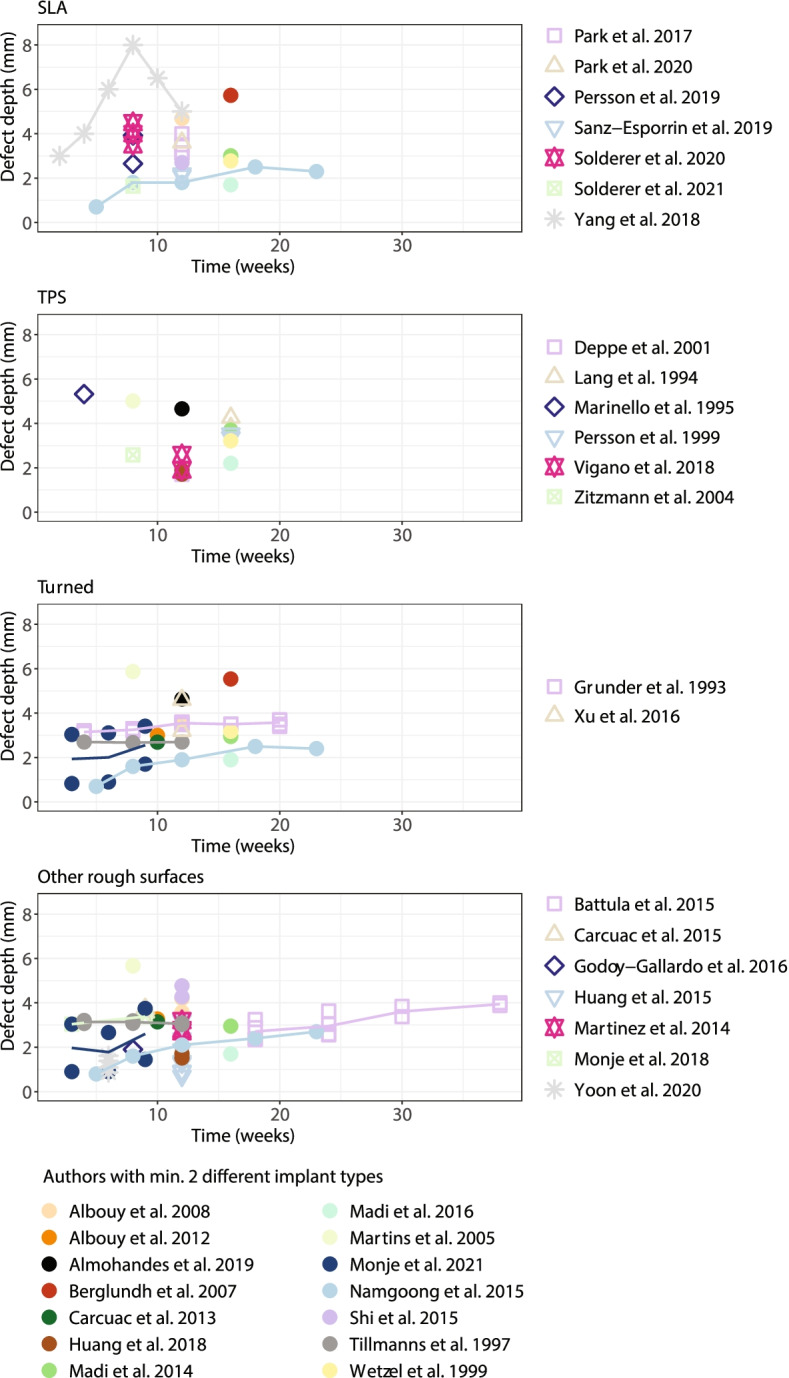
Defect depth (mm) progression over time (weeks) of all included studies split into four implant surface types: SLA, TPS, turned and other rough surfaces

All studies investigating implant type at multiple time points were identified to examine the effect of ligature application over time on defect depth development. In some studies, longitudinal data over weeks were provided, while in others, the focus was on shorter periods. An overview of all assessed studies according to defect depth development over time is given in Fig. 2.

The minimum and maximum defect depth range measured varied significantly among studies and time frames. Most observations were recorded for the duration of ligature-induced breakdown of 12 weeks (*n* = 16), followed by 8 (*n* = 10) and 16 weeks (*n* = 7) (Table 2).

**Table 2:** Range of the defect depth values at weeks 8, 12 and 16 of peri-implantitis induction

Defect depth	Week 8	Week 12	Week 16
Lowest value	**1.6 mm** [39]	**0.7 mm** [29]	**1.7 mm** [33]
Highest value	**8.0 mm** [50]	**5.0 mm** [50]	**5.5 mm** [23]

#### Defect depth development over time for implant surfaces

**Fig. 3:**
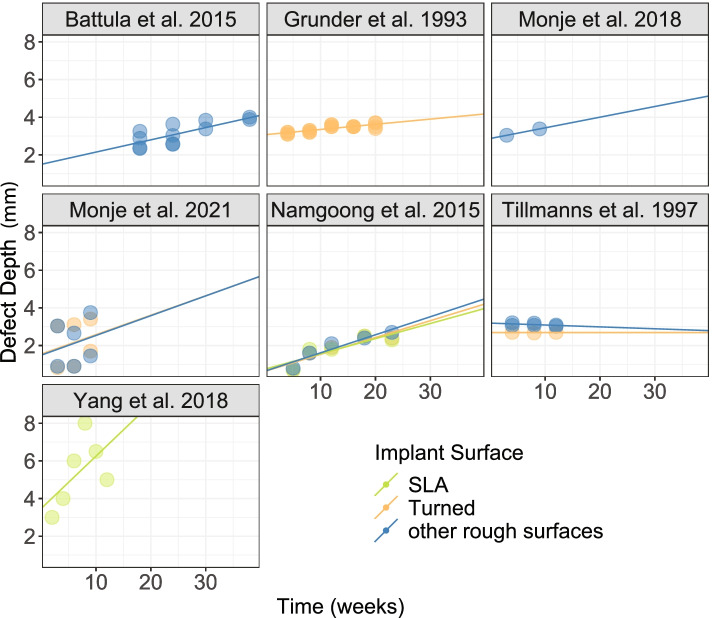
Defect depth (mm) progression over time (weeks) of seven included studies and three implant surface types

The separate simple regression models calculated for the different implant surfaces are shown in Fig. 3. In six out of seven studies, an increase in defect depth over time was observed. At the same time, a slight decrease over time was observed in one study [46]. The influence of active breakdown time seems to differ broadly. An average additional defect depth of 0.08 mm (min: −0.01 mm, max: 0.28 mm) per week was estimated.

#### Influence factors on defect depths (using measurements after 12 weeks)

**Fig. 4:**
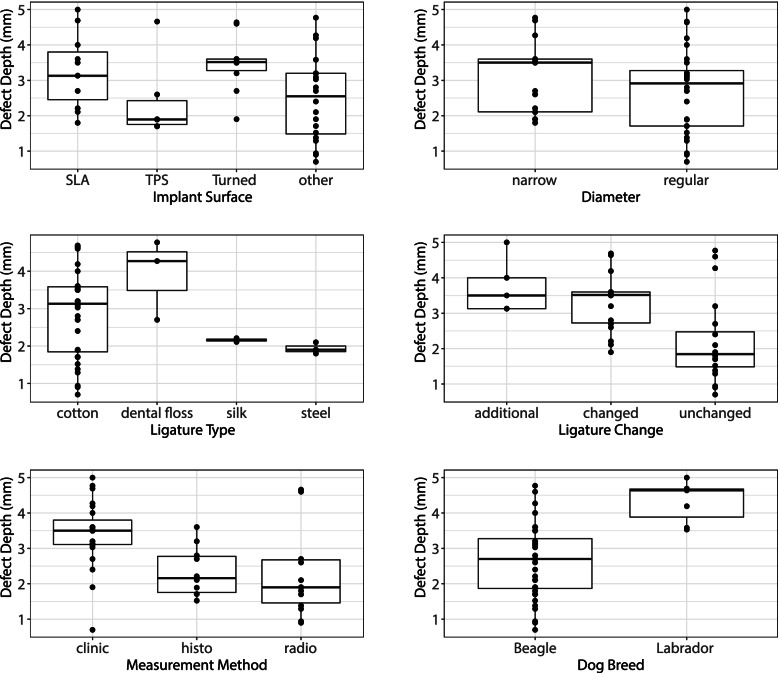
Box and whisker plot of the defect depth (mm) of the four included implant surfaces SLA, TPS, turned and other rough surfaces, implant diameter, ligature type, ligature change, measurement method and dog breed at week 12

The different distributions of measured defect depths are shown in Fig. 4, according to different selected key factors after 12 weeks of peri-implantitis induction. Presumably, large differences (e.g. between implant types, ligature type, ligature change, measurement method or even dog breed) appeared at first sight. However, these differences could not be corroborated by our multidimensional statistical analysis. Not a single key factor could be shown to influence defect depth significantly because all coefficients were shrunk to zero within our lasso regression approach. This result is likely due to (1) some groups within a factor seem different but have limited data points, thus providing little evidence, and (2) factors could not easily be disentangled, despite the multidimensional modelling because of considerable overlap and confounding (e.g. Labrador dogs with their high defect depths were all treated with cotton ligatures, possibly increasing the average defect depth of the cotton group).

##### Implant surface

Defect depths at 12 weeks of experimental peri-implantitis were described in 16 studies. A total of 12 studies referred to turned implants, 14 studies to SLA, 12 to TPS and 15 to other rough surfaces.

##### Ligature protocols

After osseointegration of the various implants, ligatures were applied around each implant and forced into a submucosal position to ease plaque accumulation. The time of ligature application varied greatly among the studies. Overall, measurements were recorded at weeks 2, 3, 4, 5, 6, 8, 9, 10, 12, 16, 18, 20, 23, 24, 30 and 38. The twelfth week had the most measured observations (*n* = 47). Different ligature materials were used. Most of the 36 studies used cotton ligatures [5, 19–21, 23–26, 28–30, 32, 34–36, 40, 42, 45, 46, 49, 52, 53]. Silk [22, 27, 31, 33, 37, 38, 43, 48, 51], steel [39] and dental floss [44] were used in other studies. The ligature material used was not recorded in two studies [47, 50]. Furthermore, the frequency of the ligature change was recorded. In the group ‘changed’, the ligatures were changed and replaced with a new after some time [19–21, 23–25, 28, 32, 35, 36, 38, 41–43, 47, 52, 53]. The group ‘unchanged’ [5, 22, 26, 27, 29, 30, 39, 44, 45, 51] did not change the ligature that was inserted first, and the group ‘additional’ added multiple ligatures to the existing one [31, 34, 37, 40, 48, 50].

##### Measurement methods

Radiographic, clinical and histological examinations or combinations were performed before peri-implantitis induction and at different time points after the induction, illustrating the resulting bone loss measured in millimetres. The defect depths were clinically assessed in 17 studies [5, 19, 22, 28, 29, 31–34, 37, 38, 40, 44, 46, 48–51]. Clinical measurements were taken by measuring the peri-implant pocket probing depth (PPD) at baseline (before ligature placement) and at different time points during the progression. The distance from the gingival margin to the bottom of the clinical sulcus was measured using a periodontal probe and described as the probing depth [28]. The clinical attachment levels (CAL, distance from the fixed point in the abutment shoulder to the bottom of the sulcus/pocket) were also measured in some studies [29, 31, 34]. Clinical bone loss during open flap surgery was recorded in some studies with a periodontal probe [32]. Different implant sites were measured in the studies, such as distobuccal, mid-buccal, mesiobuccal, mesio-lingual, mid-lingual and disto-lingual.

For radiographic analysis, standardised radiographs at ligature placement and at different time points during the progression were taken in 18 studies [20, 21, 23–27, 29, 38, 39, 42, 45, 47, 49, 51–53]. The exposure parameters and focus film distance varied among studies. Bone loss in the included studies was measured using different methods. The marginal bone height percentage, the distance from the implant margin to the marginal bone crest, was calculated in one study after radiographs at different implant sites were taken. The percentage for this value was calculated by dividing the marginal bone height by the total implant length, and this value was then multiplied by 100 [28]. The vertical bone loss, as the distance between the implant-abutment interface (IAI) and the first bone-implant contact (fBIC); ridge loss as the vertical measurement from the ridge to the IAI; ridge-fBIC as the vertical measurement from the ridge to the fBIC, representing the depth of the infrabony defect; and horizontal bone loss as the distance from the peri-implant ridge to within 3 mm of the implant body were measured in other studies [20, 21, 27, 29, 30, 33, 40, 44]. Images using computed tomography (CT) have been obtained in some studies [27, 37, 44]. In one study [37], the Aurora Tracking System (NDI, Waterloo, CA) was used to measure horizontal bone loss, representing the distance between the implants and the buccal/lingual bony plates.

Histological measurements were made in six studies [30, 31, 35, 36, 41, 43]. The dogs were anaesthetised, followed by euthanasia without pain. During surgery, the mandibles were removed, and tissue blocks containing one implant and surrounding soft and hard tissues were prepared. The samples were prepared for histologic and photomorphogenic analyses using a microscope. Different methods have been used in many studies to calculate the resulting bone loss. Bone loss was measured in some studies by determining the percentage of bone-to-implant contact (%BIC), characterising the length of the implant surface between the most coronal contact point of the implant with the bone and the apical end of the implant [22]. The histologic probing depth was measured in other studies by calculating the distance between the alveolar bone and the probing tip [31].

The data at 12 weeks after peri-implantitis induction are shown in Fig. 4. No significant difference was detected between the measurement methods.

##### Implant diameter

Implants with regular diameters (>3.5 mm) were assessed in 17 studies, and narrow diameter (<3.5mm) was also evaluated in 17 other studies. No significant difference in defect depth could be seen between the different implant diameters at 12 weeks of peri-implantitis induction.

##### Breed

Mostly Beagle dogs (29 studies) were included, followed by Labrador (eight studies), American Foxhound (two studies) and Mongrel dogs (two studies, incl. Hound dogs).

## Discussion

Several preclinical peri-implantitis studies conducted in canine models were included in this systematic review. All bone defects were ligature-induced, allowing an undisturbed plaque accumulation, leading to inflammation with progressive bone loss.

The included studies followed exceptionally heterogeneous study protocols. Few observations were made after the same period of ligature application. Due to this inhomogeneity, it is not possible to conduct meta-analytical modelling. Nevertheless, several exploratory analyses were performed. However, due to the imbalance in the values of the observations, the results should be interpreted with caution. The separate linear regressions calculated for each study that measured bone defect over time showed that the considerable range of bone defect deformation factors varies.

Four groups were formed out of all described implant surfaces in the studies: turned, SLA, TPS and ‘other rough surfaces’. Because the variable time of ‘passive progression’ has been measured in just a few studies, the periods measured have significantly differed. Therefore the authors decided to focus on the active breakdown period. Several things were shown in advance by the graphical analysis (Fig. 2). First, only a few studies have measured the defect depth of the same implant at multiple time points, resulting in difficulties in analysing and modelling the effects of time on defect depth.

Furthermore, the number of implant types investigated was highly unevenly distributed across the studies. No clear comparisons can be made between the effects of individual implant surfaces on defect depth. However, TPS implants seem to develop the lowest value of defect depth during the first 12 weeks of ligature application (Fig. 4). This should be treated with caution because of the inhomogeneous and low-value dataset. Nevertheless, this might indicate that TPS surfaces possibly cause less bone loss in preclinical peri-implantitis models. However, further investigation would have to be pursued to prove this.

Focusing on the primary question of how much bone loss can be expected after ligature-induced peri-implantitis, the values after 8, 12 and 16 weeks of ligature-induced inflammation are summarised in Table 2, representing the time points with the most data.

The progression of the defect depth caused by 8 weeks ranges from 1.6 mm [39] and 8 mm [50]. After 12 weeks, the lowest defect depth measured was 0.7 mm [29] and the highest was 5 mm [50]. The recorded defect depth after 16 weeks ranged between 1.7 mm [33] and 5.54 mm [23] (Table 2). In this outcome, the highest values measured were in the first 8 weeks, indicating a fast initial bone loss progression at the beginning of ligature-induced peri-implantitis. However, the data of Yang et al. [50] should be taken with care due to the only non-linear development of defect depths, presenting an accentuated decrease of defect depths from weeks 8 to 12. The authors did not provide any explanations for this observation. An overall marginal bone loss of 2.295 mm in preclinical animal models without mentioning a timeframe of peri-implantitis induction was shown in a recent study by Reinedahl et al. [14].

Based on data from seven studies [22, 28, 37–39, 46, 50] reporting data from multiple time points, the overall progression of inflammation was calculated with a mean additional defect depth of 0.08 mm (min: −0.01 mm, max: 0.28 mm) per week of ligatures in place. As for secondary outcomes, which included the influence of implant diameter, dog breed, ligature material and ligature change, it was impossible to determine any difference among the groups. Again, this is related to the heterogenicity and incompleteness of the included data. In Fig. 4, which may, at first sight, seem like substantial differences between the subgroups, it cannot be sustained by statistical data. Some groups contained limited observations, and little or sporadic evidence was provided.

Regarding ligature material, Reinedahl and co-workers concluded that the most marginal bone loss was produced by cotton ligatures, followed by steel, dental floss and silk ligatures [14]. This conclusion cannot be drawn from the data assessed in the current review. Cotton ligatures were used in most studies (*n* = 22), showing heterogeneous data, while only limited observations were made for the other materials, making a statistical comparison difficult. Moreover, a possible influence of ligature change on marginal bone loss was mentioned, assuming that the anaerobic flora is disrupted by the ligature change, which is more critical for continuous bone loss [14]. Once more, after this systematic assessment, no significant difference could be found between changed, unchanged and added ligatures. A discrepancy between the two studies is indeed by only including canine models in this study, whereas Reinedahl et al. had other animal models.

The Beagle dog represents the most frequently used model concerning the included dog breeds. This might be related to their obedience and size, allowing the examinators to have more dogs using less space.

Examining the influence of the diet provided for the experimental dogs was impossible due to the lack of variety among studies. Soft food for their animals was provided in the majority of the studies (*n* = 19). There were no reports about food quality in many studies (*n* = 15). Only two studies used moistened animal foods. Standard pellet food was reported in only one study [39]. The detailed food quality was often not specified, making it impossible to report a possible influence of food quality on the extent of peri-implant inflammation and bone loss.

The main strength of the present systematic review is the collection of a plethora of data from different studies, including many variables and giving a comprehensive overview of the preclinical peri-implantitis canine model. As an implication for future research, it is recommended to provide a standard deviation for the defect depth to allow comparable data analysis to enable other data analyses for researchers. Also, a universal study setting guiding experimental studies to collect their data could lead to more comparable studies. In this recommended study setting, it could be determined in which time frames observations should be made, which parameters should be measured and with what methods bone loss should be measured.

Finally, it can be stated that with all the different implant types inserted in patients nowadays, it is crucial to be aware of their impact on the surrounding bone. After extracting more information from existing experimental studies, it is still impossible to answer the question of which implant types are more preservative and which are more prone to cause an infection in preclinical settings. Therefore, more research is needed to answer this question.

Hopefully, an opportunity for future studies to compare data in peri-implantitis research and provide guidelines for a comparable study design will be provided by this systematic review. Consequently, there will be less need for future animal studies that discuss only bone loss due to experimental peri-implantitis. Future trials assessing different treatment strategies or preventive measures of peri-implantitis could be seen among the data generated in the present review.

## Supplementary Information

**Additional file 1.** Database(s): Ovid MEDLINE(R) and Epub Ahead of Print, In-Process & Other Non-Indexed Citations and Daily

## Data Availability

All generated data are included in the published paper. Raw data can be retrieved from the corresponding author.
