# Supplementary material for: Bone defect development in experimental canine peri-implantitis models: a systematic review
Source: Syst Rev. 2022 Sep 21;11:202. doi: 10.1186/s13643-022-02075-3 (PMC9494778; doi:10.1186/s13643-022-02075-3)
Supplement: Supplementary file 1 — Additional file 1. Database(s): Ovid MEDLINE(R) and Epub Ahead of Print, In-Process & Other Non-Indexed Citations and Daily [file 13643_2022_2075_MOESM1_ESM.docx]

**Additional file**

| Database(s): **Ovid MEDLINE(R) and Epub Ahead of Print, In-Process & Other Non-Indexed Citations and Daily** | | |
| --- | --- | --- |
|  | | |
| **#** | **Searches** | **Results** |
| 1 | (exp Dental Implants/ or ((dental or oral or tooth or teeth) adj3 (implant* or prosthes*)).ti,ab.) and (ligation/ or (ligature* or ligation* or constriction* or "plaque accumulation" or "mechanical overload" or "bacterial inoculation").ti,ab.) | 313 |
| 2 | (Peri-Implantitis/ and (experimental* or induced).ti,ab.) or ((experimental* or induced) adj3 (periimplantiti* or peri-implantiti*)).ti,ab. | 205 |
| 3 | 1 or 2 | 424 |
| 4 | Bone Resorption/ or osteoly*.ti,ab. or ((bone or osseo* or osteo* or tissue) adj3 (loss or resorption or lost or height or level)).ti,ab. or defect*.ti,ab. | 561050 |
| 5 | 3 and 4 | 231 |
| 6 | limit 5 to medline | 213 |
| 7 | 6 not (humans not animals).sh. | 140 |
| 8 | 5 not 6 | 18 |
| 9 | 8 not ((human* or patient*) not (animal* or simulat* or dog* or mice or murine or mini-pig* or pig* or swine or porcine or rat or rats or rabbit*)).mp. | 9 |
| 10 | 7 or 9 | 149 |
